# Commentary: A proteome-wide atlas of humoral immunity to *Mycobacterium tuberculosis* across the spectrum of disease

**DOI:** 10.3389/fimmu.2026.1970659

**Published:** 2026-10-05

**Authors:** Samreen Fatima

**Affiliations:** Department of Immunology and Infectious Diseases (IID), Harvard T.H. Chan School of Public Health, Boston, MA, United States

**Keywords:** antibodies, correlates of protection, humoral immunity, proteome microarray, tuberculosis, vaccine antigens

## 1 Introduction

For most of the past century, antibodies were treated as bystanders in tuberculosis- incidental markers of exposure but not mediators of protection (1, 2). The humoral evidence that might redirect the effort is itself genuinely mixed. Passive transfer of polyclonal antibody has protected only inconsistently (2), whereas defined monoclonals against *Mycobacterium tuberculosis* (*Mtb)* surface antigens can enhance opsonization and restrict intracellular growth (3, 4), lower bacterial burden *in vivo* (5, 6), and block dissemination (7). The genetic picture is similarly split: antibody- and Fc-receptor-deficient mice control *Mtb* less effectively (8, 9), yet people lacking IgG show no clear deficit in control (2). Moreover, antibodies, plasma cells, and Fc-receptor-bearing effector cells are abundant in the *Mtb* granuloma (10, 11); *post-hoc* analysis of the MVA85A trial linked higher Ag85A-specific IgG to reduced disease risk (12); and B-cell depletion in macaques increased lesional bacterial burden (13). These findings collectively kept the humoral question open while leaving its targets, specificities, and mechanisms in humans largely undefined.

More recent work has sharpened the case that, where antibodies do contribute, they act largely through Fc-dependent effector mechanisms, whether dissected by systems serology, Fc-engineering, or passive transfer (14–17). It is against this unresolved backdrop that the study by Khan et al. (18) becomes a landmark of scope: using a near-whole-proteome microarray (~4,000 proteins), the authors map IgG reactivity across ATB, LTBI, IGRA-resistant “resisters” (RSTRs), and rhesus macaques given protective intravenous versus non-protective intradermal BCG. The result is the first high-resolution atlas of humoral targeting across the full spectrum of *Mtb* exposure and control.

The strengths of the study include its careful framing of IgG responses as either mediators or surrogate markers, as well as its explicit acknowledgement of optimistic cross-validation, modest cohort sizes, compartment mismatch, and platform limitations (18). In addition, the authors move beyond binding by validating a subset of hits using recombinant proteins and functional assays (18).

## 2 Binding, function, and protection are not interchangeable

Khan et al. acknowledge that the precise role of antibodies- mechanistic or surrogate, remains unresolved (18). The additional point here is that the study’s individual findings can be assigned to different evidentiary levels, which lead to different claims. At the first level, the proteome microarray establishes antigen-specific binding. IgG reactivity identifies distinct antigen sets in RSTRs versus LTBI, increased binding to selected antigens after IV versus ID BCG, and a set of 12 antigens including LpqN, PPE14, EchA10, Rv2100, and Rv2721c, that remain enriched across LTBI, RSTR, and IV-BCG groups after multiple-testing correction (18). These observations identify antigens that are immunogenic and associated with phenotypes of Mtb control, but binding alone does not establish antibody effector activity.

At the second level, Khan et al. provide functional evidence for a smaller subset of antigens. In RSTRs antibodies specific for DevS, SodA, and LpqH showed enhanced monocyte phagocytosis, SodA- and LpqH-specific antibodies showed enhanced neutrophil phagocytosis, and DevS- and LpqH-specific antibodies showed enhanced complement deposition relative to LTBI (18). These results move those antigen-specific responses beyond binding to measurable Fc-dependent function, but they still do not demonstrate that the responses cause protection *in vivo*. Then they define the third level- the causal contributor of protection. This level would require an intervention that changes infection or disease outcome such as antigen-specific passive transfer, depletion or blockade, or vaccination followed by challenge. The present study does not test that level directly. Thus, the key advance of this three-level framework is not simply to repeat the authors’ caveat, but to specify which claims in the atlas are supported as binding associations, which are supported by functional assays, and which remain candidate protective targets requiring causal validation.

## 3 What does convergence across “protected” groups mean?

The most striking claim is that LTBI, RSTRs, and IV-BCG macaques converge on a shared set of antigens (12 significant after correction, including LpqN, PPE14, EchA10, Rv2100, and Rv2721c) (18). This convergence identifies antigens associated across populations with enhanced Mtb control but does not by itself establish that the shared antigens are causal protective targets. These three states are immunologically dissimilar, so the shared binding need not reflect a shared protective mechanism. An alternative reading is that the signature is partly one of antigen accessibility: the Gene Ontology analyses repeatedly return membrane and secreted proteins, the very antigens most available to B cells, and the authors themselves attribute this to proteome redundancy (18, 19). A convergent focus on surface and secreted proteins may therefore track what the immune system can see as much as what protects.

## 4 Reading the negative vaccine-antigen result

The finding that 8 of 11 clinical vaccine antigens bind at or below background in protected populations (18) is provocative and represents a well-reported, valuable negative result. It should nonetheless be interpreted in the light of the platform’s limitations. The array expresses polypeptides in an *Escherichia coli* cell-free system that omits glycosylation and may not reconstitute conformational epitopes (19). For antigens such as ESAT-6, which behaves as a molten globule with little stable tertiary structure (20), or the secreted Ag85 mycolyltransferases (21), low array signal may reflect under-representation rather than true non-immunogenicity — a caveat the authors concede for ESAT-6 and Ag85B (18). “Poorly immunogenic on this array” and “poorly immunogenic *in vivo*” are not equivalent statements. This distinction is especially important as targeted vaccine studies have shown protective efficacy for some of these antigens, in small animal models (22).

## 5 An IgG atlas may underrepresent other antibody isotypes

A subtler tension sits inside the paper’s own data. The discovery screen is IgG-only, yet the authors’ systems serology analysis identifies IgM, IgA, and Fc-receptor-binding features among those distinguishing RSTR from LTBI (18), highlighting the broader importance of antibody isotype and Fc-dependent functional diversity in shaping humoral immunity (23, 24). Moreover, robust IgM responses have been associated with prevention of Mtb infection following intravenous BCG vaccination in rhesus macaques (25). This is an association rather than evidence that IgM itself mediates protection. Nevertheless, an IgG-centric proteome map may underrepresent antibody isotypes associated with favorable outcomes in one of the cohorts used to anchor the atlas’s protected phenotype. Multi-isotype arrays are an obvious and tractable next step.

## 6 Discussion: from associations to mechanisms

Several complementary experiments would convert this atlas from a map of associations into a map of mechanisms. Longitudinal sampling could establish whether resister antibodies precede or merely accompany the phenotype (26). Multiantigen vaccines or monoclonals against the top convergent antigens, Fc-engineered and passively transferred into FcγR-humanized mice (8, 15, 27), together with mycobacterial growth-inhibition assays (28), would test whether these antigen-specific responses make a causal contribution to protection. Furthermore, re-testing vaccine antigens in native, glycosylated form, and extending profiling to HIV-positive (29) and pediatric (30) populations and to compartment-matched samples, would address the platform and generalizability caveats.

Nevertheless, Khan et al. have built a resource that reframes the question from *whether* antibodies target *Mtb* to *which* targets matter and *in whom*. Its value lies less in assigning protection to any single antigen than in providing a framework for prioritizing candidates while keeping binding, measurable antibody function, and causal protection as distinct evidentiary levels.
